# Revealing higher than expected diversity of Harpacticoida (Crustacea:Copepoda) in the North Sea using MALDI-TOF MS and molecular barcoding

**DOI:** 10.1038/s41598-019-45718-7

**Published:** 2019-06-24

**Authors:** S. Rossel, P. Martínez Arbizu

**Affiliations:** 1 Senckenberg Research Institute, German Centre for Marine Biodiversity Research (DZMB), Südstrand 44, 26382 Wilhelmshaven, Germany; 2 Marine Biodiversity Research, Institute for Biology and Environmental Sciences, Carl von Ossietzky University Oldenburg, Oldenburg, Germany

**Keywords:** Mass spectrometry, Proteomic analysis, Biodiversity, Zoology

## Abstract

The North Sea is one of the most extensively studied marine regions of the world. Hence, large amounts of molecular data for species identification are available in public repositories, and expectations to find numerous new species in this well-known region are rather low. However, molecular reference data for harpacticoid copepods from this area in particular but also for this group in general is scarce. By assessing COI barcodes and MALDI-TOF mass spectra for this group of small crustaceans, it was discovered that there is a huge unknown diversity in this area. In total, COI sequences for 548 specimens from 115 species of harpacticoid copepods are presented. Over 19% of these were new to science and ten MOTUs were found to be part of cryptic species complexes. MALDI-TOF mass spectra were assessed for 622 specimens from 75 species. Because results were in concordance with species delimitation by COI barcoding and also enabled recognition of possible cryptic species, the discriminative power of this technique for biodiversity assessments is highlighted. Findings imply, species diversity in this group may be largely underestimated and total species number can be expected to be much higher than previously assumed.

## Introduction

Over the last few years, DNA barcoding, as defined by Hebert *et al*.^1^, based on a fragment of the cytochrome *c* oxidase subunit I (COI), has become a broadly used technique for specimen identification. In data depositories such as GenBank^2^ and BOLD^3^, scientists store molecular data for numerous plant and animal species, which can be used to identify specimens around the globe. But even though COI barcoding of metazoan species is a broadly used technique to reliably identify specimens, this technique is rarely used for harpacticoid copepods. However, using COI barcoding combined with comprehensive libraries would be a huge advantage and accelerate difficult and time consuming identification^4–6^ of harpacticoid species. Moreover, diversity not noticed due to morphological misidentification of very similar species disregards certain evolutionary and ecological aspects because neither does close morphological similarity come along with identical ecology nor with the same genetic background^7^. Nevertheless, only few studies collecting barcode data for reference were conducted on these small crustaceans. Even if included, they often only make up a small part of the collected data^8–12^.

Thus, the vast diversity of harpacticoid copepods is by far not represented in the public gene depositories. Currently (September 27, 2018), GenBank finds only about 739 sequences for the search term “Harpacticoida COI OR Harpacticoida CO1 OR Harpacticoida COX1”, of which 178 are attributed to only two species: *Tigriopus californicus* (Baker, 1912) (n = 117) and Cletopsyllidae sp. (n = 61). The remaining sequences are distributed across 98 identifications to species level, 57 identifications to genus level, 47 identifications to family level and 24 identifications to order level only. In BOLD, the query “Harpacticoida” returns 870 published records of which 382 are not identified to species level. Of these, 194 are not further determined than to order level (accessed: February 13, 2019). At the same time, undescribed species diversity is estimated to over 30,000 species^13^ and several cases of cryptic diversity or species complexes have already been revealed by molecular studies^14–16^. This implies an even higher cryptic or pseudo-cryptic diversity and hence, a diversity that was not noticed morphologically. Nevertheless, many recent studies focusing on meiofauna still mainly employ morphological specimen identifications^17,18^.

An alternative method for rapid specimen-by-specimen identification to the expensive and comparably time-consuming COI barcoding is Matrix-Assisted Laser Desorption/Ionization Time-of-Flight Mass Spectrometry (MALDI-TOF MS). For analysis, analytes are embedded into a matrix preventing them from defragmentation by high radiation during ionization using a laser^19^. Mass detection of proteins subsequently results in a proteomic fingerprint widely used in microbiology to identify bacteria, viruses or fungi^20–22^. Predominantly in pilot studies, MALDI-TOF MS was also employed to identify metazoan taxa like nematodes^23^, insects^24–27^, fish^28^ or by Laakmann *et al*.^29^ and Bode *et al*.^30^ for calanoid copepods. Successful identification of smaller harpacticoid copepods was already shown by Rossel and Martínez Arbizu^31,32^. Amongst others, the authors emphasized the importance of sediment sample storage at low temperatures for MALDI-TOF MS applications^32^.

With the current study, morphological identifications combined with DNA barcoding and MALDI-TOF MS were used to receive insights into the diversity of benthic copepods from the North Sea. For the first time widely supported by molecular methods in one of the most extensively studied and well known marine areas in the world^12^. By providing COI-barcode and MALDI-TOF mass spectra libraries for copepods from three orders (Harpacticoida, Canuelloida and Cyclopoida), this study aims to show that at least for meiofauna diversity, there is a huge gap in knowledge. Thus, a higher diversity than expected is uncovered with regard to cryptic diversity of these highly abundant crustaceans based on congruent data from COI barcoding and MALDI-TOF MS data. Subsequently, large reference libraries for use in future biodiversity assessments employing DNA-based methods such as metabarcoding or MALDI-TOF MS-based assessments are provided.

## Results

### COI barcoding

**Figure 1:**
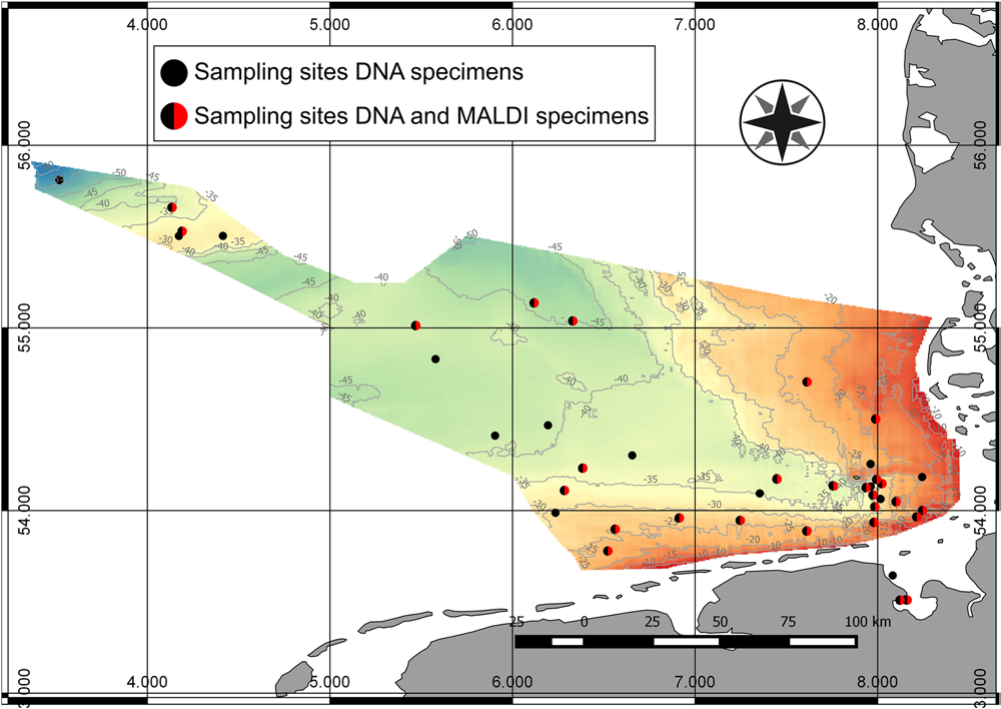
North Sea sampling sites. Bathymetric map of the German exclusive economic zone (EEZ) in the North Sea depicting sampling sites for DNA specimens (black) and MALDI-TOF MS specimens (red). The map was generated with QGIS^71^ (v. 2.18.27, http://qgis.org).

**Figure 2:**
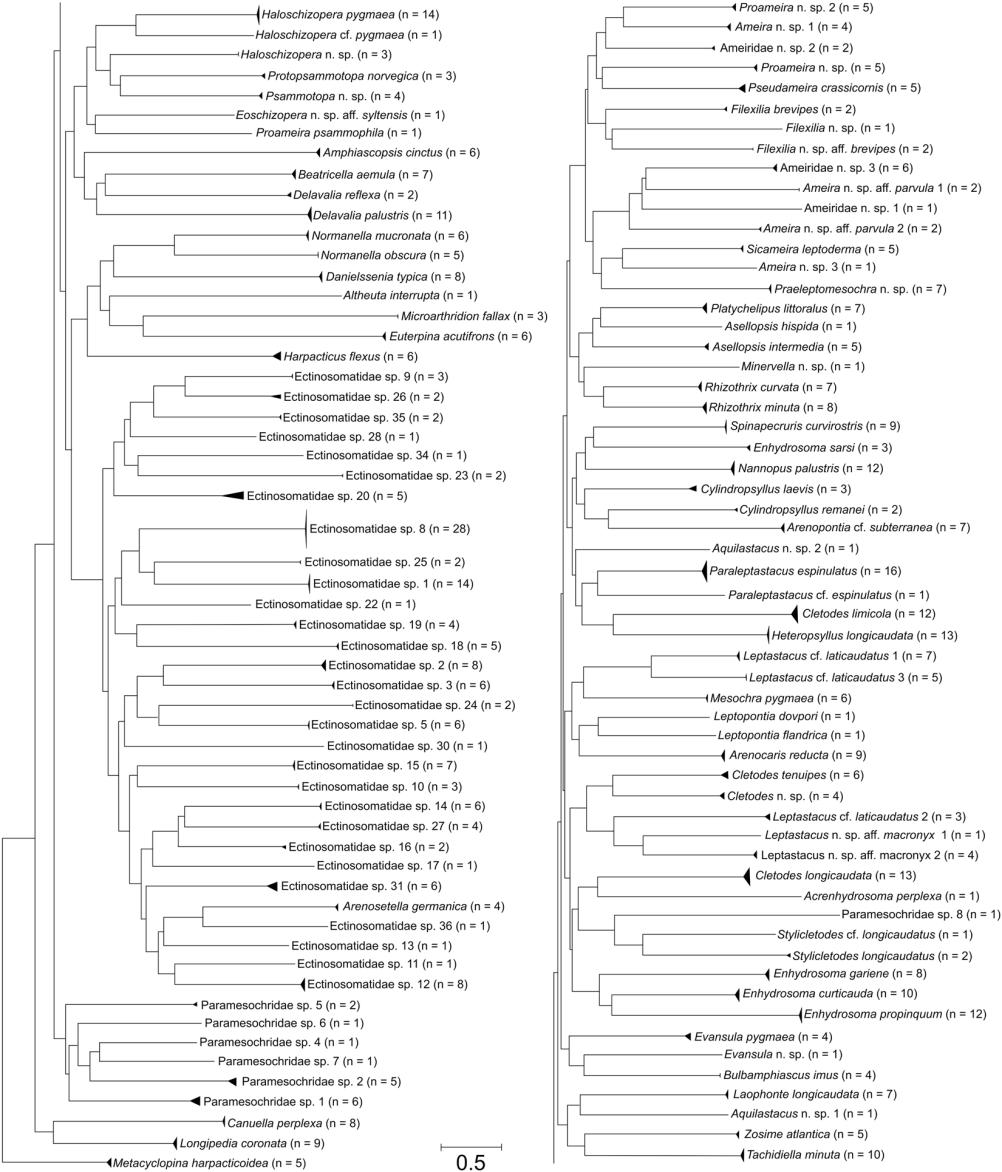
Neighbor-Joining tree based on 548 COI sequences and K2P distances. Branch lengths indicate genetic distances. The tree presents the 115 MOTUS that were also recognized by ABGD with *M. harpacticoidea* (Cyclopoida) as outgroup to Canuelloida and Harpacticoida. Numbers in brackets behind species names give numbers of sequences per taxon.

**Figure 3:**
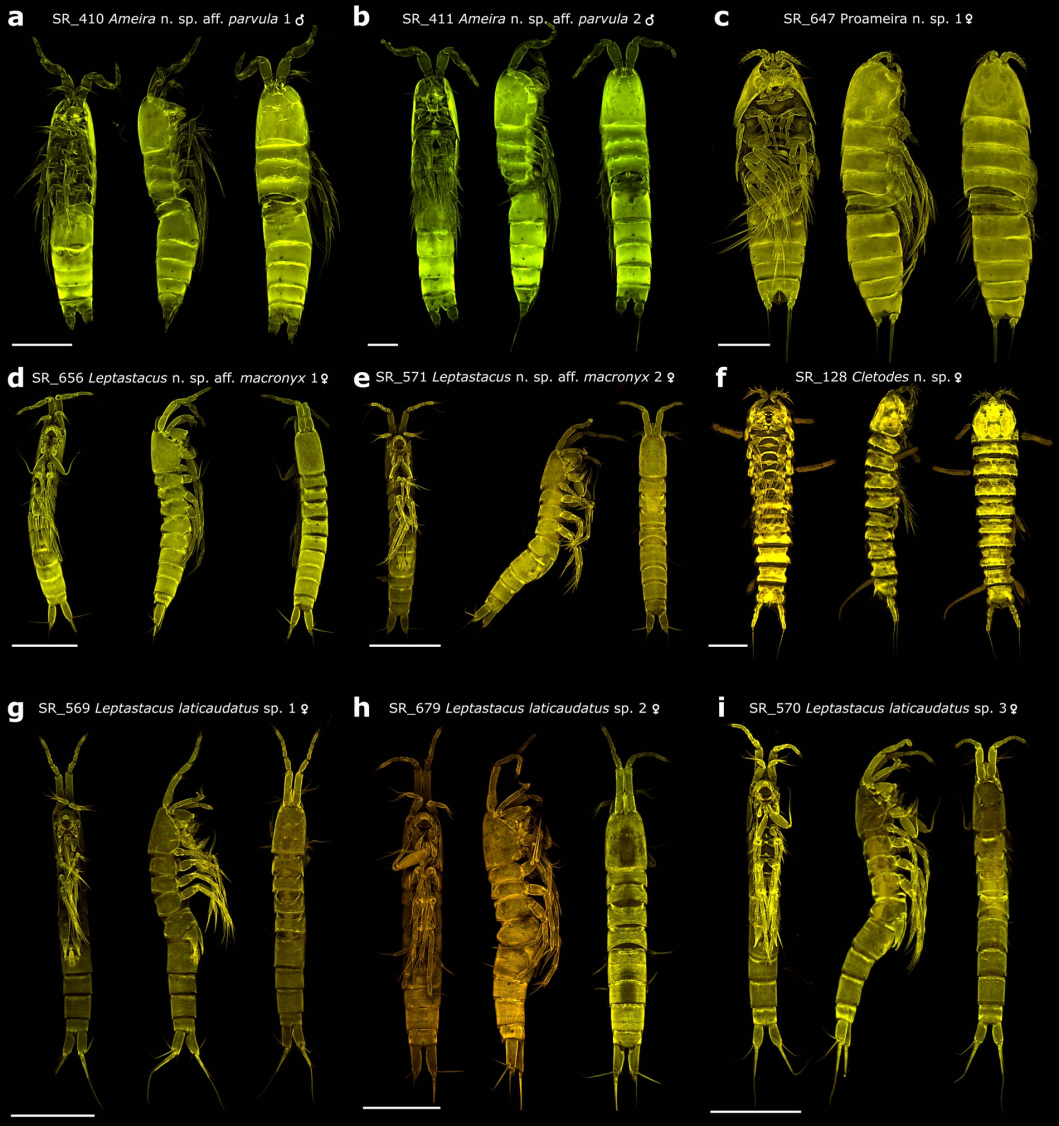
Maximum projection confocal laser scanning microscopy images. Scale bar for all specimens equals 100 µm. **(a**–**f)** Images of some of the species discovered in this study as new to science. **(g**–**i)** Images of female specimens of the *Leptastacus laticaudatus* cryptic species complex.

From 41 stations in the North Sea (Fig. 1), 548 COI consensus sequences of species from three copepod orders (Cyclopoida: 1 species; Canuelloida: 2 species; Harpacticoida: 112 species) were assessed. These are distributed over 25 families, 48 genera and 115 molecular operational taxonomic units (MOTUs) (Fig. 2) of which 100 were not previously available in BOLD or GenBank. The number of sequences per MOTU ranged from 28 for Ectinosomatidae sp. 8 to only one for 30 MOTUs (singletons: species/MOTUs represented by a single consensus sequence). Of the 115 MOTUs, 79 were identified to species level prior to DNA analysis, recognizing 22 as new to science (Fig. 3a–f, Supplementary Figs S1–3).

**Figure 4:**
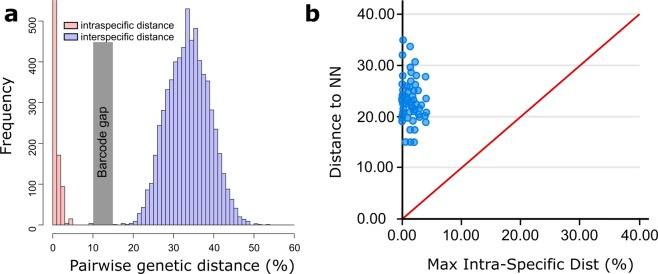
Genetic distances plots. (**a**) Plot depicting intraspecific (red) and interspecific K2P (blue) distances, resulting in the barcoding gap (grey) of the provided dataset. (**b**) Maximum K2P intraspecific distances versus nearest neighbor distances as calculated by BOLD sequence analytics. The graph clearly depicts the distances to the nearest neighbor (NN) to always be distinctly higher than the maximum intra-specific distance. Distances crossing the red line would indicate distances to be higher within species than to the nearest neighbor and hence would present these to be inappropriate for specimen identification.

Because different sets of forward and reverse primers were used, producing larger fragments than the standard barcode fragments (see methods section), sequence lengths ranged from 373 to 896 base pairs. For *Alteutha interrupta* (Goodsir, 1845) an insert of 3 bp length was found. Furthermore, inferring from the final amino acid alignment, for *Microarthridion fallax* Perkins, 1956 30 bp deletions and 69 bp insertions without stop codon were found. Hence, this does not seem to be a nuclear mitochondrial DNA segment (NUMT) but an actual COI sequence. Intraspecific K2P distances ranged from 0 to 9.31% and interspecific congeneric distances ranged up to 34.59%. Total interspecific distances ranged from 15.32% to 53.02%. No overlap of highest intraspecific and lowest interspecific distance (Fig. 4a) or of distance to the nearest neighbor to maximum intraspecific distance (Fig. 4b) was found. All identification errors during the simulated specimen identification scenario using SpeciesIdentifier 1.8^33^ under the Best Match (BM) criterion occurred in singletons. *Paraleptastacus* cf. *espinulatus* was considered ambiguously identified and incorrect identifications were found for 29 (5.29%) sequences. By the Best Close Match (BCM) criterion, 94.52% of the submitted sequences were correctly identified. Singletons were considered incorrectly identified during identification scenario and eight specimens were without a match any closer than the 5% cutoff threshold calculated by SpeciesIdentifier. The All Species Barcodes (ASB) approach identified 87.95% of the sequences correctly while again 38 specimens were without match closer than the 2.27% threshold. No specimens were incorrectly identified with ASB, whereas all specimens from species with only two available specimens were considered ambiguously identified.

Among the 22 species new to science (Supplementary Table T1), several species were identified by different identification keys as *Ameira parvula* (Claus, 1866) (Fig. 3a,b, Supplementary Fig. S1). None of these specimens were attributable to the original description and clearly differed from each other morphologically and genetically. Morphological identification of two leptastacid MOTUs led to *Leptastacus macronyx* (Scott T., 1892) (Fig. 3d,e) but specimens did not match the original description of this species either. Furthermore, evidence for cryptic species was found for *Leptastacus laticaudatus* Nicholls, 1935 with three different MOTUS assigned to the morphospecies (Fig. 3g–i). Similarly, for *P. espinulatus*, *Haloschizopera pygmaea* (Norman & Scott T., 1905) and *Stylicletodes longicaudatus* (Brady, 1880) each, one additional morphologically identified specimen with deviating COI sequence was found. The remaining 36 morphologically unidentified MOTUS belonged to the Paramesochridae (6 MOTUs) and Ectinosomatidae (30 MOTUs). The sequences submitted to BOLD were automatically assigned to 122 BOLD Barcode Index Numbers (BINs) of which 15 existed before in other projects. None of the BINS showed discordances in BOLD BIN discordance analysis.

**Figure 5:**
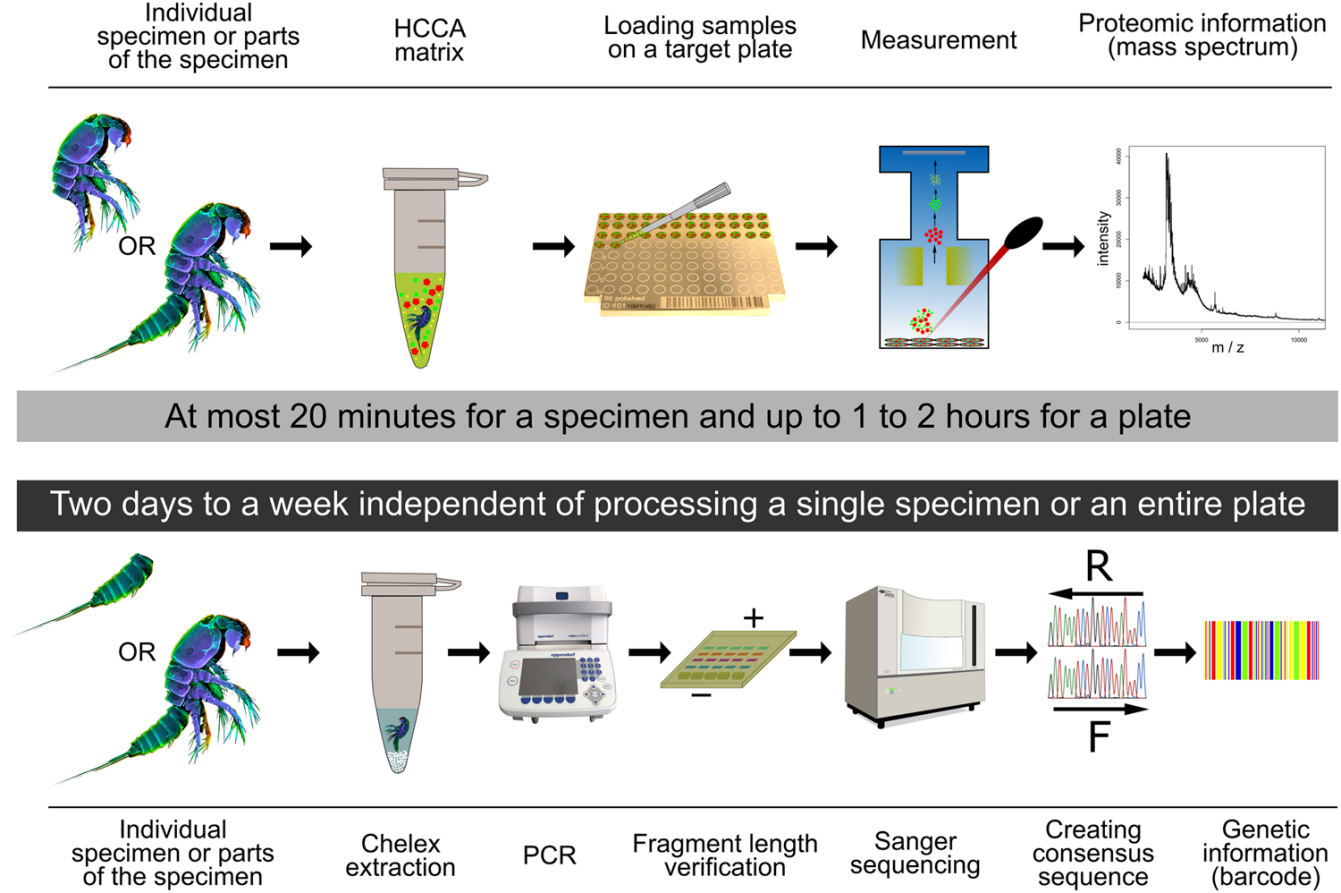
Workflow comparison MALDI-TOF MS and COI barcoding. The upper part illustrates the workflow for MALDI-TOF MS. A single specimen is incubated in up to 5 µl of HCCA matrix for 5 minutes. The resulting solution is transferred onto a single spot of a 96-spot target plate and measured in a mass spectrometer to receive a proteomic fingerprint. From specimen to mass spectrum this only takes around 20 minutes to 1 or 2 hours if an entire plate is processed and measured. To receive a barcode, a DNA extraction is carried out. The DNA extract is then used for PCR and amplification is checked by gel electrophoresis. As sequencing is often carried out at external sequencing facilities, it can take several days up to a week from specimen to final barcode. To obtain a barcode and simultaneously a mass spectrum from a single specimen, it has to be cut into two parts, processing one part for MALDI-TOF MS and one for DNA barcoding.

**Figure 6:**
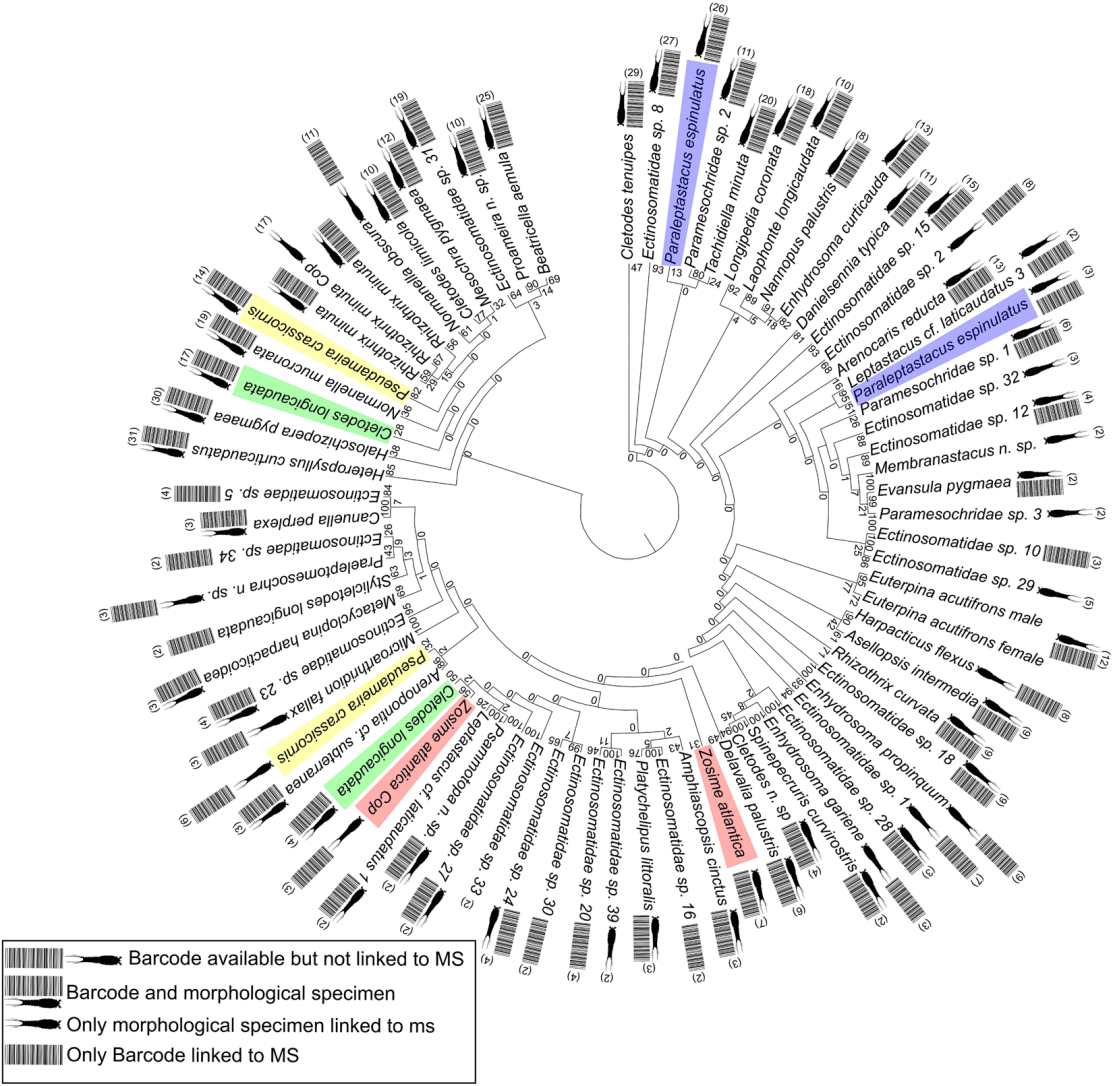
Tree from the cluster analysis carried out on 612 MALDI-TOF mass spectra. It was calculated with 1,000 bootstrap replicates. Numbers in brackets indicate number of specimens measured per species. Clusters containing specimens used in simultaneous measurement of a mass spectrum and sequencing of a COI barcode are marked with a barcode sign next to a copepod sign. Colored clusters are species found in more than one cluster. All *P. espinulatus* (blue) and *C. longicaudata* (green) clusters are directly linked to COI sequences by specimens analyzed simultaneously by both methods. Morphology of *P. crassicornis* (yellow) specimens and juveniles of *Z. atlantica* (red) was re-examined after measurement but identification did not change. Clusters could also not be linked to different sexes or sampling sites but rather to degraded MS signal.

For 161 of the 548 specimens analyzed by COI barcoding, a mass spectrum was measured simultaneously to support the species delimitation based on the proteomic fingerprint by morphological identification and molecular classification likewise (see Fig. 5 or methods for detailed explanation). These 161 specimens belonged to 57 of the 115 discovered MOTUs (Supplementary Table T1). For further twelve species a mass spectrum was linked to a COI sequence due to matching species available from former studies^32^ or due to specimens from same samples with matching morphology (only *Normanella obscura* and *Praeleptomesochra* n. *sp*.) (Fig. 6).

### MALDI-TOF mass spectrometry

A reference library containing 622 mass spectra with specimens from 75 species is provided. These are distributed over three copepod orders, 22 families and 36 genera (Fig. 6). Except for nine of these 75 species and operational taxonomic units (OTUs) (*Bulbamphiascus imus* (Brady, 1872), Ectinosomatidae sp. 3, Ectinosomatidae sp. 4, Ectinosomatidae sp. 14, *Enhydrosoma sarsi* (Scott T., 1905), *Minervella* n. sp., *Paraleptastacus* cf. *espinulatus* Nicholls, 1935, *Platichelipus littoralis* Brady, 1880 juvenile, *S. longicaudataus* sp. 1), at least two spectra from conspecific specimens were available. Only six species are provided without corresponding COI sequence (Ectinosomatidae sp. 29, Ectinosomatidae sp. 32, Ectinosomatidae sp. 33, Ectinosomatidae sp. 39, *Membranastacus* n. sp., Paramesochridae sp. 3).

Species from the two families Paramesochridae and Ectinosomatidae were not identified to species level morphologically. When neither morphological identification was applied, nor a DNA sequence could be linked to a group of mass spectra, the results of clustering supported by a Random Forest (RF)^34^ analysis were used to delimitate species. This was only applied to five species (Ectinosomatidae sp. 29, Ectinosomatidae sp. 32, Ectinosomatidae sp. 33, Ectinosomatidae sp. 39 and Paramesochridae sp. 3). Four species (*Cletodes longicaudata* Brady & Robertson D., 1875, *P. espinulatus*, *Pseudameira crassicornis* Sars G.O., 1911 and *Zosime atlantica* Bodin, 1968) were found in two different clusters each (Fig. 6, colored clusters). In *C. longicaudata* and *P. espinulatus*, both clusters were supported by specimens, for which simultaneously a barcode was obtained. The second *Z. atlantica* cluster referred to juvenile copepods (Fig. 6, red colored) only. Aside from this species, juvenile specimens were only measured for *Rhizothrix minuta* (Scott T., 1903). But in this species an additional cluster was found within the main species’ cluster and not separated from this. The only species in this analysis that shows a species cluster divided into male and female is *Euterpina acutifrons* (Dana, 1847). To support the clusters found by hierarchical clustering, an RF analysis was conducted. Of the 582 specimens included to train the RF model, twelve were assigned to different classes resulting in an error of 2.06%.

## Discussion

All specimen identification scenarios conducted in SpeciesIdentifier 1.8 resulted in good success and most species were unambiguously identified. Under the BM criterion, the 29 singletons resulted in 5.29% incorrect classifications. A further singleton, *P*. cf. *espinulatus*, was designated as ambiguously identified because equally small distances were found to different species. With the BCM and ASB criterion, 38 MOTUS were without match closer than the threshold calculated by SpeciesIdentifier. This rather indicates that the threshold of 2.27% inferred by SpeciesIdentifier is not applicable to all species than an actual incapability of the sequence to serve in specimen identifications since intraspecific distances were found to range up to 9.31%. Ambiguously identified sequences recognized by the ASB criterion referred to species with only two specimens in the dataset. This however happened because of the software premise that a returned query sequence has to be followed by two sequences of the same species, which was not possible for these.

Most of the 15 shared BINs (identical or very similar sequences) in BOLD are from projects in the North Sea or the adjacent Atlantic Ocean. Specimens of *Longipedia coronata* Claus, 1862 from the North Sea shared a BIN with some specimens of *Clytemnestra scutellata* Dana, 1847 from Norway. For this species, only an image of a juvenile copepod is submitted to BOLD which makes morphological comparisons difficult. It implies however, either COI barcodes to be unsuitable to resolve between these unrelated species or the occurrence of misidentification. For *Evansula pygmaea* (Scott T., 1903), sequences accessible via BOLD and GenBank did not match with the data collected in this study. This again facilitates potential difficulties of morphological identification or cryptic species within this copepod group. Furthermore, specimens of *E. acutifrons* from the current study shared a BIN with *E. acutifrons* sequences from the Atlantic Ocean off the coast of Spain as well as off the coast of Greece in the Mediterranean Sea. The concordance in COI barcodes supports a widespread distribution of this holoplanktonic species^35^. Moreover, one ectinosomatid species recorded here from shallow coastal waters shares a BIN with identical sequences from Ectinosomatidae off the Canadian Pacific coast with a beeline distance of over 7,700 km. Although transoceanic distribution of meiofauna species was shown before^36^, these identic COI sequences would be the first genetic evidence for such a wide distribution of a shallow water Harpacticoida. But morphological examinations and multi-locus molecular analyses would be necessary to invest if these sequences actually refer to the same species.

Besides large scale distribution of single species, the COI data gives evidence for several cases of small scale species complexes and possible cryptic species. Two MOTUs from the family Ameiridae were identified by most identification keys as *Ameira parvula*. However, using Wells’ very comprehensive identification key^37^, a final identification as *A. parvula* was not possible as, even though many final character combinations were given, none fitted the examined specimens perfectly. Aside from these, two further MOTUS were recognized belonging to the genus *Ameira* without satisfying specimen identification and, depending on the applied identification key, with classifications as *A. parvula* as well. Notably, none was completely in concordance with the original description of this species and all of these species were morphologically distinguishable from each other. Such morphological variation was however not observed for three MOTUs identified as *L. laticaudatus*. Female specimens of all three MOTUs were repeatedly morphologically examined. All of them were congruent (e.g. setal formula, setation leg 5 etc.) with the re-description of this species by Huys^38^ and within the variation described for the arrangement of small spinules on the last abdominal segment and the caudal rami. Also, the length-width ratio of the caudal rami was within the described range of *L. laticaudatus* and variability of the distal process of the P5 between the three MOTUs was not observed. Sequence diversity, on the other hand, was high comparing *L*. cf. *laticaudatus* 1 to *L*. cf. *laticaudatus* 2 (29.9%) and even higher comparing *L*. cf. *laticaudatus* 2 to *L*. cf. *laticaudatus* 3 (31.15%). These clearly exceed the intraspecific variations generally described for crustaceans^12,39^. In the NJ tree, *L*. cf. *laticaudatus* 2 furthermore clusters apart from the remaining two MOTUS with *L. macronyx*. Comparing *L*. cf. *laticaudatus* 1 to *L*. cf. *laticaudatus* 3, sequence divergence was considerably lower (15.32%), still exceeding classical limits of intraspecific variation. Interestingly, these two morphologically identical MOTUs were obtained from the very same sample implying a possible sympatric speciation or secondary sympatry after a speciation event. For *P. espinulatus* and *S. longicaudatus* each, two MOTUS were retrieved. Because specimens were cut into two body parts and used also for MALDI-TOF MS, a re-examination of the specimens from these singleton MOTUs was not possible. Specimens of the *L. macronyx* complex (Fig. 3d,e) were distinguishable from each other, but could not satisfyingly be associated to any described species.

Cryptic species and species complexes among harpacticoid copepods were observed or suspected various times based on molecular evidence for different taxa such as *Microarthridion littorale* (Poppe, 1881)^40,41^, *N. palustris*^14^, *Schizopera*^15^, *Cletocamptus deitersi* (Richard, 1897)^16^ or the *Zausodes*-complex^42^. In some of these taxa, high genetic differences were found for species suspected to be in morphological stasis. Nevertheless, repeatedly species records were published, giving morphological variations of known species from locations other than the type location without describing these as species new to science. This accounts not only for pre-molecular analysis times like for *N. palustris*^43^ but also for more recent publications (e.g. *M. littorale*^44^). This contradicts findings of molecular studies but also ignores results of crossing experiments in which different populations of the cosmopolitan species *Phyllognathopus viguieri* (Maupas, 1892) were already described to show different mating behavior^45^ and to be completely reproductively separated^46^. Specimens from different spatial populations did not interbreed and therefore likely constituted sibling species rather than one biological species. Such records contribute to the underestimation of harpacticoid diversity as morphological differences are ignored and potentially undescribed species are assigned to existing ones. Although some molecular studies contradict the idea of widely distributed, shallow water meiofauna species, other authors provided data on species with broad distribution ranges, supported by both morphological and molecular analyses^47,48^.

Besides original descriptions, Wells’ comprehensive identification key^37^ also includes re-descriptions and, in some cases, species records with a descriptive character. This leads to various possible character combinations to identify certain species. The cosmopolitan species *A. parvula*, for which several MOTUs were found, for example, is represented in Wells’ key^37^ by 15 different variations. Likewise, 17 different combinations lead to *Sarsamphiascus minutus* (Claus, 1863) (listed in the key as *Amphiascus minutus*) and *N. palustris* is also represented with many varying character combinations. Especially in cases of high species throughput, this may lead to misidentifications as basically any closely related species may be assigned to one of the given character combinations. Therefore, new species with divergent morphology to the original description are simply overlooked and falsely assigned to a species, hence receiving broader distributions than true or expected. As, for example, *A. parvula* is frequently recorded from different habitats all around the globe such as the deep sea^49^ or different coastal areas^50–52^. Its wide distribution might also base on an overlooked diversity, driven by hasty identification using morphological keys without supporting these with molecular information. Hence, and because the provided data shows that high morphological similarity does not refer to similar genetic background, this study emphasizes the use of molecular methods, such as COI barcoding in biodiversity assessments.

For the North Sea, Huys *et al*.^53^ carried out the hitherto most comprehensive, morphological biodiversity assessment on benthic copepod, identifying 278 copepod species of which 121 (43.5%) were found new to science. Taking this for scale, in the current study it was possible to collect COI sequences for 41.37% of the species expected for the entire North Sea within the comparably small area of the German Exclusive Economic Zone. To date, there is no knowledge about the actual species number or the species composition of harpacticoid copepods from this area, especially not supported by genetic information. After Huys *et al*.^53^ no comprehensive assessments for the North Sea were carried out. Because molecular techniques were not widely available yet at that time and most of the newly discovered species were not described, comparisons of recent findings to that former study are hardly possible. Since then, studies either focused on small area assessments^54^ with only a few species or meiofauna studies with identifications to a higher taxonomic level than species^55^, but always exclusively morphologically. Also, only a few new species from this area were described over the last decades^56,57^, therefore the actual diversity of the North Sea Harpacticoida still remains unknown.

Studies on harpacticoid diversity supported by molecular markers are scarce and only few of them are dedicated to this taxon exclusively. Some are limited to only few species^58^, others frequently reveal a high species and cryptic diversity^14,15,42^ that was not noticed before. This highlights the demand for integrative species descriptions containing molecular information such as the COI barcode next to the morphological description. Although frequently requested, only few descriptions are supported by any genetic information^59–61^. However, the scarce knowledge about the genetic background of widely distributed species and the increasing knowledge on cryptic species in Harpacticoida indicate the need for more assessments of molecular data for reference purposes to enable broader studies on species distribution and connectivity. This would also prevent putative morphological confusions or overestimations of species distributions. In this study, a total of 115 species were detected in a rather small part of the North Sea. Of these, at least 19% could not be assigned to already described species and 8.7% were part of potentially cryptic species complexes probably containing further undescribed species. Hence, we are only at the beginning of closing this unsuspected gap in knowledge on harpacticoid diversity and species distribution in this part of the North Sea. Nevertheless, by supporting these findings with easily accessible molecular data, further studies may lead to a more complete image of harpacticoid diversity in this area, which seems to be less well studied than thought before.

In addition to COI, mass spectrometry data was collected on 26.98% of benthic copepod species reported by Huys *et al*.^53^ for the entire North Sea. Proteomic OTUs are well supported by RF as well as cluster analysis and are furthermore congruent with species delimitation based on COI fragment. Taxa that are morphologically difficult to identify such as Ectinosomatidae and very small Paramesochridae, which are comparable in size to small deep-sea species, were found in distinct clusters and hence can be identified rapidly using MALDI-TOF MS. Species from the same genus (e.g. *Cletodes*, *Normanella* and *Enhydrosoma* among others) were identified without ambiguous clustering or classification by RF. Moreover, species from the putative cryptic species complex *Leptastacus* cf. *laticaudatus* were shown to be separated confidently based on protein mass spectra.

Specimens from different ontogenetic stages were observed in separate clusters in *R. minuta* and *Z. atlantica*. Interestingly, *E. acutifrons* specimens were separated into male- and female-specific clusters. Since this was not observed for any other species, storage and size might have had an influence on results. In fact, *E. acutifrons* is a comparably large species and therefore measurements might result in a mass spectrum providing more information. Furthermore, of the twelve specimens analyzed for this species, eleven were processed on the day of sampling. Therefore, no potential adverse effects of storage as they were described by different authors^26,32,62,63^ applied to these specimens. However, the clustering results of some examined specimens were impacted by adverse effects of sample storage. Ambiguous clustering in cases of *P. espinulatus*, *C. longicaudata* and *P. crassicornis* were not attributable to sex, life stage or sampling site. For *P. crassicornis* the mass spectra from these two clusters differed in quality. Although specimens from both clusters were obtained from same samples, storage seems to have impacted specimens differently. Samples from the smaller cluster showed mass spectra with lower intensities, stronger noise and fewer peaks on average (87.29 vs. 72.67). Therefore ambiguous clustering of this species is attributed to mass spectra quality. The same can be found as potential explanation for *P. espinulatus* and *C. longicaudata* as mass spectra from the clusters with fewer specimens were noisier and less intense. Nevertheless, as various specimens show overlapping, equally aberrant signals, these mass spectra are important for libraries thought to be used for further specimen identification from real samples, where specimens may be partly degraded as well.

In RF analysis, *Amphiascopsis cinctus* (Claus, 1866) was the only species with a class error of one. All specimens were assigned to other classes implying that this species’ class was not well characterized by RF based on submitted mass spectra, although all spectra showed good intensities and only little noise. For one specimen (SRM_3251), the amount of measured mass peaks decreased by 30 compared to the other specimens. This might have resulted in an unstable RF class. Also, this RF class was based on only three specimens of this species, supporting the idea that it was not possible to sufficiently characterize this species class.

The overall good identification success using MALDI-TOF MS is in line with results from other authors^30,64^. Some studies have also observed clustering based on different ontogenetic stages^29,64^ however, clusters based on different sexes of the specimens are only rarely reported^65^. This, again, highlights the potential power of this technique to identify unknown specimens for example in biodiversity research. Concerns about the influence of gut contents on the ability to use mass spectra to identify specimen in small analyzed species could not be confirmed yet. In several studies, hundreds of specimens from different sampling sites were analyzed and specimens of the same species still showed distinct species-specific signals^30,31,64,66^ even though food resources in different locations might be different. Additionally, the amount of peptides and proteins from the gut might be too low, to be detected by this technique at all. However, in larger animals such as biting midges where gut contents make up large parts of the entire biomass, changes in mass spectra were observed but without influence on the markers important to identify the species^63^. Moreover, MALDI-TOF MS is a lot cheaper than COI-barcoding with less than 0.50 € (0.57 $) per sample and provides almost immediate results (compare Fig. 5). It is an interesting method for taxa with difficult morphological identifications such as small copepods or important disease vectors such as mosquitos^67^ and fleas^68^. Especially in biodiversity assessments, traditionally favoring quantitative data over qualitative data, this technique can be used as a cost-effective, yet fast alternative to genetic barcoding to identify animal groups occurring in high abundances or taxa which are difficult and time-consuming to identify due to their small size^29–32,64,69,70^.

## Conclusion

With this study focusing on harpacticoid copepods, it was shown that even an extensively studied area like the North Sea still bears a lot of unknown diversity. Actual diversity of this group is largely underestimated by the inability of recognizing morphological differences in possible cryptic species. In this context, MALDI-TOF MS as an emerging tool for species identification of metazoan species and COI-barcoding both were shown to be capable of delimiting a large number of species morphologically difficult to discern and to reveal possible cryptic species diversity. Hence, if molecular techniques were employed in more studies, libraries would not only serve faster identification of specimens in future studies but also lead to better comparability of results over time and between studies.

This is highly important as the inability to recognize the actual diversity leads to underestimation of meiofauna diversity and hence also to underestimation of the importance of meiofauna research and of the role of meiofauna. Consequently, studies on meiofauna need to be supported, besides morphological identifications, by molecular data to ensure comparability over time. The results from this study largely contributed to the growth of genetic data for these benthic crustaceans in public data repositories.

## Material and Methods

### Sampling, specimens processing and storage of vouchers

Samples were taken from 41 sampling sites across the German EEZ from coastal areas, the area around Helgoland to sites such as the Dogger Bank in a distance of 300 km to the coast (Fig. 1). The map was generated with QGIS^71^ (v. 2.18.27, http://qgis.org) using public data from the project Geopotenzial Deutsche Nordsee (http://www.gpdn.de). Sediment samples were collected on different cruises between March 2014 and March 2017 with the research vessels Heincke (HE417, HE432 and HE447) and Senckenberg (IBR-1 and IBR-2) using a Multiple Corer (MUC) or Van Veen Grab Sampler. Further samples were collected by hand from the littoral zone in 2017. Detailed sampling information of all genetic specimens can be found at the Barcode of Life Data system^3^ in the project ‘NSHX North Sea Harpacticoida 2′ (Code: NSHX). Sediment samples were initially fixed with 96% undenatured ethanol and postfixed after 24 hours. Samples from the Heincke cruises were stored at room temperature (RT) while all other samples were stored at −25 °C until further processing. Sediment samples were sieved through a 40 µm sieve before density-gravity-centrifugation using Kaolin and Levasil® (Kurt Obermeier GmbH & Co. KG, Bad Berleburg, Germany) was applied^72^.

Single specimens were separated, morphologically identified using a Leica DMRE microscope and stored in 96% undenatured ethanol at −25 °C until further processing. To morphologically identify specimens, different identification keys and original literature were used^13,37,73^. Some species were only identified to family level as literature on these families is too scarce to ensure valid identifications (Ectinosomatidae and Paramesochridae). Many original descriptions of ectinosomatid species are stated as being unprecise or incorrect^74,75^ and paramesochrid species are among the smallest harpacticoids which may lead to unrecognized misidentifications. Identified specimens of *Harpacticus flexus* did not match the original description by Brady & Robertson D., 1873 but the description of *Harpacticus flexus* from the North Sea by Mielke^76^ with only one inner seta at the second segment of the second pair of legs.

After extraction of DNA or peptides and proteins, cuticles were, if retained, stored in 70% ethanol at Senckenberg German Centre for Mar Biodivers Research (DZMB, Wilhelmshaven, Germany). Specimens used to simultaneously extract DNA and proteins were cut into prosome and urosome body parts and cuticles were not retained.

### DNA isolation, PCR amplification and sequencing

DNA was extracted from whole specimens using either NucleoSpin Tissue Kit (Macherey-Nagel, Düren, Germany) following manufacturer’s protocol or InstaGene matrix (Bio-Rad Laboratories, Munich, Germany) in a volume of 20 µl. To extract DNA using InstaGene matrix, samples were placed in a thermocycler for 50 minutes (min) at 56 °C and 10 min at 96 °C. In case of simultaneous extraction of DNA and proteins, only the prosome body part was used to extract DNA from. Because amplification of the barcoding fragment was not possible by using universal Folmer primers^77^ only, different combinations of forward and revers primers were used (Table 1) in a vapo.protect Mastercycler pro S Cycler (Eppendorf, Hamburg, Germany) using AccuStart II PCR ToughMix (QuantaBio, Beverly, Massachusetts, USA). Detailed information on forward and reverse primer combination for each specimen can be found in Supplementary Table T1. For some specimens, reverse primer Coxr2^78^ was used which extends the amplified fragment beyond the classical barcode length, thus amplifying fragments with lengths of up to 1,050 bp^78^ including the entire barcode fragment. The amount of DNA ranged between 2 and 5 μl in a reaction volume of 20 μl containing 10 μl 2X AccuStart II PCR ToughMix, 0.2 μl of primers with a concentration of 20 pmol/μl, filled up to the final reaction volume with molecular grade water. Cycler amplification settings were: an initial step at 94 °C for 5 minutes, a denaturation step at 94 °C for 45 seconds (s), annealing at 45 °C for 75 s and elongation at 72 °C for 75 s. After 40 repeats of the latter three steps, a final elongation step for 2 minutes at 72 °C was carried out. Two μl of the amplified PCR products were verified for size conformity by electrophoresis in a 1-% agarose gel stained with GelRED™ using commercial DNA size standards. PCR products were purified and sequenced in both directions at a contract sequencing facility (Macrogen Europe, Amsterdam, Netherlands) using an ABI 3730xl DNA Sequencer. Sequencing was carried out using M13 sequencing primers (forward: 5′-TGTAAAACGACGGCCAGT-3′; reverse: 5′-CAGGAAACAGCTATGAC-3′) (see Fig. 5 for comparison of DNA barcoding and MALDI-TOF MS workflow).

**Table 1:** List of COI amplification primers used in this study.

PrimerName	direction	Sequence 5′-3′	Reference
LCO1490	forward	GGTCAACAAATCATAAAGATATTGG	Folmer *et al*. (1994)^77^
jgLCO1490	forward	TITCIACIAAYCAYAARGAYATTGG	Geller *et al*. (2013)^94^
Coxf	forward	GGTCCTGTAATCATAAAGAYATYGG	Cheng *et al*. (2013)^78^
HCO2198	reverse	TAAACTTCAGGGTGACCAAAAAATCA	Folmer *et al*. (1994)^77^
CopCOI2198X	reverse	GGGTGRCCRAARAATCARAA	Rossel and Martínez Arbizu (2018)^32^
jgHCO2198	reverse	TAIACYTCIGGRTGICCRAARAAYCA	Geller *et al*. (2013)^94^
Coxr2	reverse	TCTATCCCAACTGTAAATATRTGRTG	Cheng *et al*. (2013)^78^
Cop-COI + 20	forward	GACTAATCATAAAGATATTGGTAC	Chang (2007)^50^

Primer combinations for every specimen can be obtained from BOLD or Supplementary Table T1.

### Sequence alignment and data analyses

Consensus sequences were generated from forward and reverse sequencing reaction results using SeqTrace^79^. Amplification of the correct gene fragment was tested by Blast search^80,81^. The consensus sequences were aligned using SeaView^82^ and checked for stop codons indicating possible NUMTs. Resulting sequences were uploaded to GenBank and BOLD including trace files of forward and reverse reactions.

To check the provided data set for a barcoding gap, pairwise Kimura-2-Parameter (K2P) intra- and interspecific distances were computed using MEGA 6^83^. To further test applicability of the DNA library a sequence-based specimen identification scenario simulation according to Meier *et al*.^33^ was carried out using SpeciesIdentifier 1.8 software^33^. Each sequence is queried against the provided library and assigned to a species according to three different criteria: Best Match (BM), Best Close Match (BCM) and All Species Barcodes (ASB). With BM criterion, the sequence is assigned to the species it is most similar to. The BCM and ASB criteria use a user-supplied threshold for species identification simulation. All sequences with matching species only above the threshold are considered unidentified. If the query sequence is assigned to different species with equal distances below the threshold, it is considered ambiguously classified by BCM criterion. Under the ASB criterion, a barcode is queried and only considered correctly identified when query sequence is returned and followed by all (at least two) conspecific sequences with similarities below the given threshold (singletons will always be returned as incorrectly identified). Sequences only assigned to species other than the prior identification are considered false classification. The chosen threshold of 2.27% used for BCM and ASB, was calculated as the 5% cutoff of intraspecific distances by SpeciesIdentifier from the sequence alignment.

The alignment was submitted to the ABGD online application, used to delimitate species, carried out using the default settings (Pmin = 0.001; Pmax = 0.1; Steps = 10; Relative gap width = 1.5; Nb bins = 20; JC69) (http://wwwabi.snv.jussieu.fr/public/abgd/abgdweb.html)^84^. To visualize the species clusters, a Neighbor-Joining (NJ) tree was calculated using MEGA 6 with K2P distance. The resulting tree was rooted using *Metacyclopina harpacticoidea* (Klie, 1949) (Cyclopoida) as outgroup to the remaining Canuelloida and Harpacticoida.

Sequences were automatically assigned to Barcode Index Numbers (BINs) in BOLD, forming clusters that show high concordance with species^85^. This permitted a fast comparison to sequences of specimens previously submitted to BOLD and their distribution and potential spatial overlap or distance with specimens from the current study.

### MALDI-TOF MS

Individual morphologically identified specimens from IBR cruises and hand sampling were sorted and separated into 1.5 ml Eppendorf microcentrifuge tubes with a small amount of ethanol (up to 0.5 μl). Ethanol was completely evaporated at room temperature and evaporation was checked for each specimen at a dissecting microscope. To extract peptides and proteins, 4 μl of a matrix solution were added, containing α-Cyano-4-hydroxycinnamic acid (HCCA) as a saturated solution in 50% acetonitrile, 47.5% molecular grade water and 2.5% trifluoroacetic acid. After an incubation time of 5 minutes, the solution was applied to one spot on a target plate (Fig. 5) and air dried for co-crystallization of matrix and proteins and peptides.

Protein mass spectra were measured between 2k to 20k Dalton on a Microflex LT/SH System (Bruker Daltonics) using method MBTAuto with laser intensity between 30% and 40%. Peak evaluation during measurement was carried out in a mass peak range between 2k–10k Dalton using a centroid peak detection algorithm, a signal to noise threshold of 2 and a minimum intensity threshold of 600, with a peak resolution higher than 400. Proteins/Oligonucleotide method was employed for fuzzy control with a maximal resolution ten times above the threshold. To create a sum spectrum, 240 satisfactory shots were summed up. Each spot was measured between one to three times.

Mass spectra data was processed in R^86^ (version 3.2.3) using packages ‘MALDIquant’^87^ and ‘MALDIquantForeign’^88^. Protein mass spectra were trimmed to an identical range from 2,000 to 20,000 m/z and smoothed with the Savitzky-Golay method^89^. The baseline was removed based on SNIP baseline estimation method^90^ and spectra were normalized using the TIC method implemented in MALDIquant. Noise estimation was carried out using a signal to noise ratio (SNR) of 7. Peaks were repeatedly binned with the ‘binpeaks’ command from MALDIquant with a tolerance of 0.002 in a strict approach to the number of peaks for the whole dataset was reduced from 1,538 peaks to 899 peaks. The resulting intensity matrix was Hellinger transformed for further use in cluster and RF analyses.

### Cluster analysis

A cluster analysis was carried out including all species with at least two specimens. A Hellinger transformed matrix was used and analyzed using Ward’s D clustering algorithm^91^ with Euclidean distances and 1,000 bootstrap replicates. The resulting tree was exported as newick format using R package ‘ape’^92^ and further processed using Mega 6.

### Random forest analysis

To support the quality of the presented mass spectra library, a RF analysis was carried out including all species with at least three specimens using the R package randomForest^93^. Within the model, RF finds mass spectra that more likely belong to a different class based on the given data and result in a respective OOB error. To calculate the RF model, 2,000 trees were generated with 35 analyzed characters at each tree split. To prevent overfitting of highly abundant species, the ‘sampsize’ for the RF analysis was restricted to three.

## Supplementary information

Dataset 2

Dataset 1

## Data Availability

Sequences were uploaded to BOLD and GenBank (Accession-Numbers: MH670482 - MH670585; MH708076 - MH708122; MH976521 - MH976661; MK506113 – MK506116. Metadata (Sampling site, depth, sampling date and utilized primers) are stored for respective species in BOLD Project ‘North Sea Harpacticoida 2′ (Code: NSHX). Hellinger transformed data matrix containing all submitted MALDI-TOF MS spectra and respective metadata are available as a dataset on dryad (10.5061/dryad.f8s1f6m). Untransformed data prior to peak binning of mass spectra and raw data is also stored as a dataset on dryad (10.5061/dryad.f8s1f6m).
