# Supplementary material for: Revealing higher than expected diversity of Harpacticoida (Crustacea:Copepoda) in the North Sea using MALDI-TOF MS and molecular barcoding
Source: Sci Rep. 2019 Jun 24;9:9182. doi: 10.1038/s41598-019-45718-7 (PMC6591307; doi:10.1038/s41598-019-45718-7)
Supplement: Supplementary file 1 — Dataset 2 [file 41598_2019_45718_MOESM1_ESM.pdf]

Revealing higher than expected diversity of Harpacticoida (Crustacea:Copepoda) in the North Sea using MALDI-TOF MS and molecular barcoding

S. ROSSEL*[1,2] and P. MARTÍNEZ ARBIZU[1,2]

[1]Senckenberg Research Institute, German Centre for Marine Biodiversity Research (DZMB), Südstrand 44, 26382 Wilhelmshaven, Germany.

[2]Marine Biodiversity Research, Institute for Biology and Environmental Sciences, Carl von Ossietzky University Oldenburg, Oldenburg, Germany

*Correspondence: Sven Rossel, Fax: +49 4421 9475111; E-mail: sven.rossel@senckenberg.de

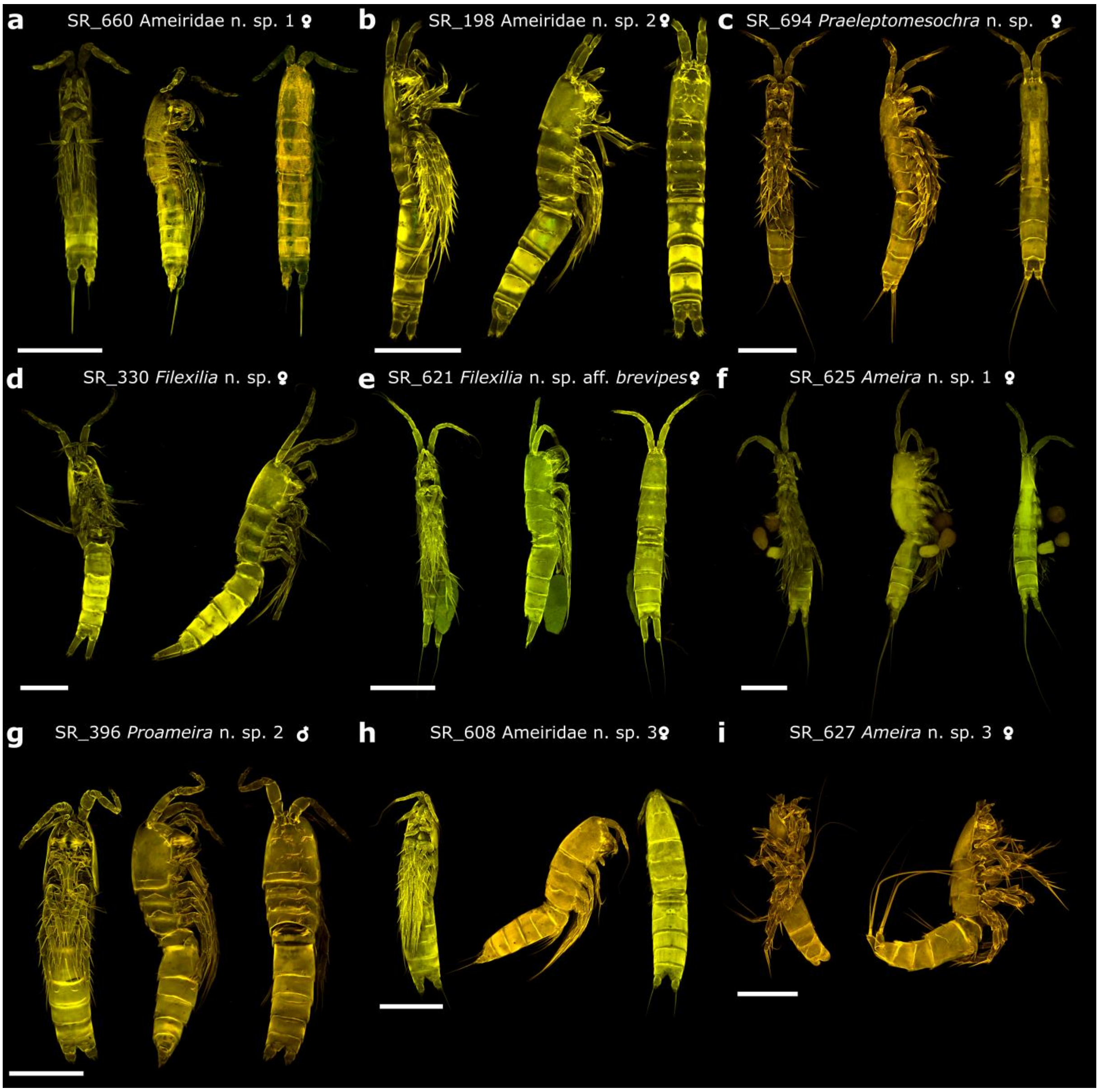

Supplementary Fig. S1: Maximum projection confocal laser scanning microscopy images of species new to science from the family Ameiridae.

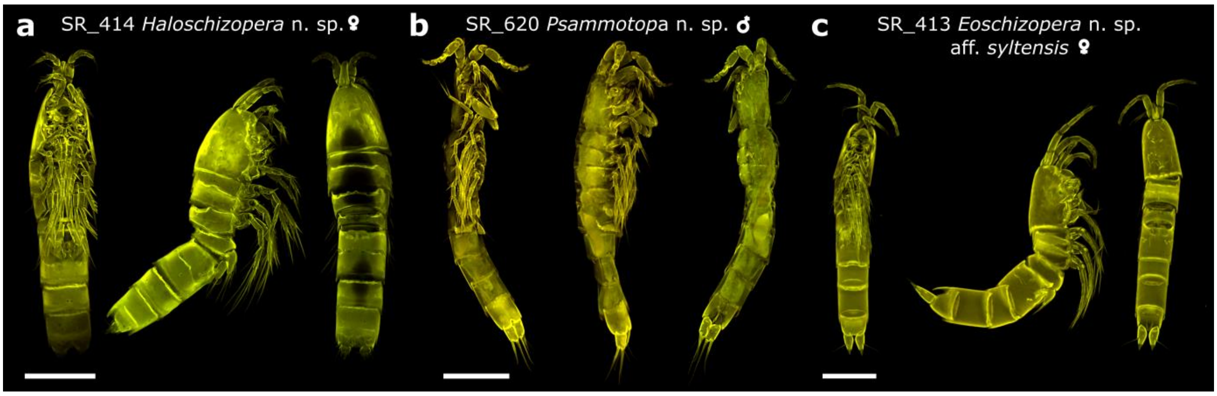

Supplementary Fig. S2: Maximum projection confocal laser scanning microscopy images of species new to science from the family Miraciidae.

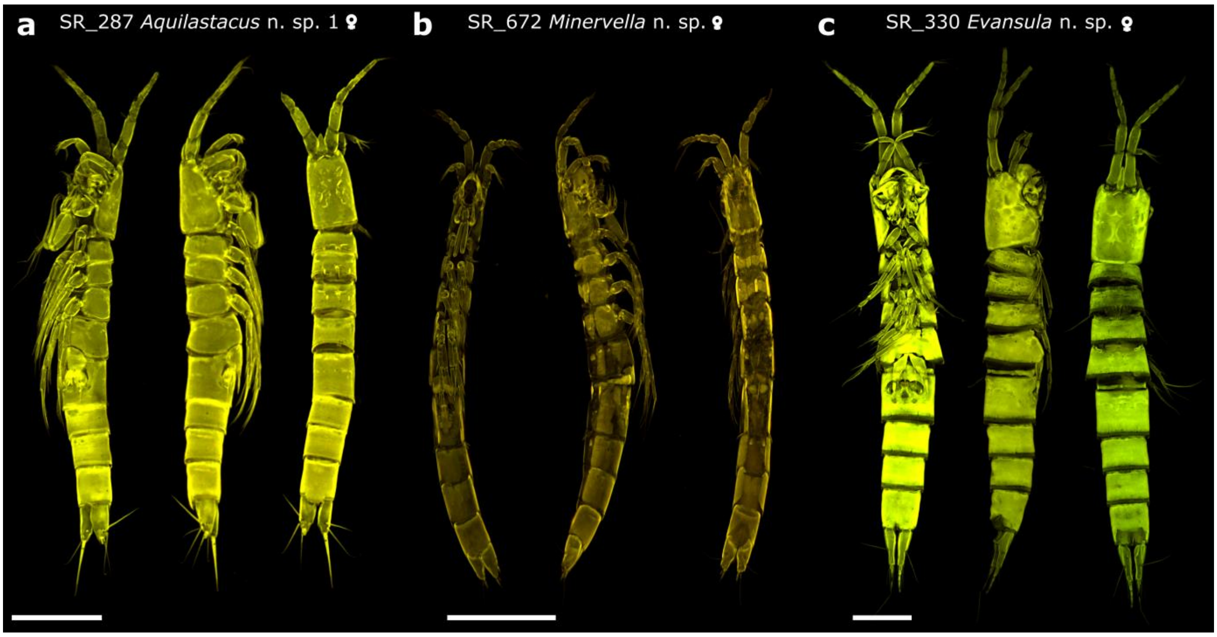

Supplementary Fig. S3: Maximum projection confocal laser scanning microscopy images of species new to science from the families Leptastacidae and Cylindropsyllidae.
